# Supplementary material for: [image]-roaming dynamics in the formation of [image] following two-photon double ionization of ethanol and aminoethanol
Source: Sci Rep. 2025 Jan 25;15:3201. doi: 10.1038/s41598-024-84531-9 (PMC11762715; doi:10.1038/s41598-024-84531-9)
Supplement: Supplementary file 1 — Supplementary Information. [file 41598_2024_84531_MOESM1_ESM.pdf]

Supplementary material for: H_2-roaming dynamics in the formation of H_3^+ following two-photon double ionization of ethanol and aminoethanol

Aaron Ngai[1], Sebastian Hartweg[1,*], Jakob D. Asmussen[2], Björn Bastian[3], Matteo Bonanomi[4,5], Carlo Callegari[6], Miltcho Danailov[6], Michele di Fraia[6], Raimund Feifel[7], Sarang Dev Ganeshamandiram[1], Sivarama Krishnan[8], Aaron LaForge[9], Friedemann Landmesser[1], Ltaief Ben Ltaief[2], Moritz Michelbach[1], Nitish Pal[6], Oksana Plekan[6], Nicolas Rendler[1], Lorenzo Raimondi[6], Fabian Richter[1], Audrey Scognamiglio[1], Tobias Sixt[1], Richard J. Squibb[7], Katrin Dulitz[10], Frank Stienkemeier[1], and Marcel Mudrich[2]

[1]Institute of Physics, Albert-Ludwigs-Universität Freiburg, Freiburg, Germany
[2]Department of Physics and Astronomy, Aarhus University, Denmark
[3]Wilhelm-Ostwald-Institut für Physikalische und Theoretische Chemie, Universität Leipzig, Germany
[4]Dipartimento di Fisica Politecnico, Milano, Italy
[5]Istituto di Fotonica e Nanotecnologie (CNR-IFN) Milano, Italy
[6]Elettra — Sincrotrone Trieste S.C.p.A., Basovizza, Trieste, Italy
[7]Department of Physics, University of Gothenburg, Gothenburg, Sweden
[8]Department of Physics, Indian Institute of Technology Madras, Chennai, India
[9]Department of Physics, University of Connecticut, Storrs, Connecticut, US
[10]Institut für Ionenphysik und Angewandte Physik, Universität Innsbruck, 6020 Innsbruck, Austria
[*]Corresponding author: sebastian.hartweg@physik.uni-freiburg.de

1 Experimental parameters

XUV pulse energies from 0 to 62 μJ, after beam-transport-induced transmission losses, were used, with spot sizes ranging from 50 to 150 μm FWHM. UV pulse energies ranged from 50-200 μJ. The specific pulse energies used for each measurement are summarized in Supplementary Table 1.

2 Data analysis

Raw data were taken by all combinations of having the pump and probe pulses blocked and unblocked. "Foreground" data is taken as the difference between pump ON probe ON and pump OFF probe ON. "Background" data is taken as the difference between pump ON probe OFF and pump OFF probe OFF. Enhancement of ion yield is defined as ("Foreground"/"Background" - 1), e.g., an enhancement=0% denotes no difference between foreground and background, and enhancement=100% denotes a foreground signal twice as large as the background signal.

To obtain averages and errors of overlapping mass peaks, we first fit the TOF spectrum with multiple Gaussians. From

Supplementary Table 1. XUV and UV pulse parameters used for datasets shown in the main text and in this supplementary material.

Species	XUV photon energy (eV)	XUV pulse energy (μJ)	UV pulse energy (μJ)
CH_3CH_2OH	31.7	~47	~176
CH_3CH_2OH	24.7	~30	~177
CH_3CH_2OH	21.1	~14	—
CD_3CD_2OD	31.7	~44	~88
$NH_2CH_2CH_2OH$	31.7	~50	~120
$NH_2CH_2CH_2OH$	21.1	~14	~130

the fitted Gaussian, we then create a masking filter for each mass peak, that describes which portion of the ion signal can be ascribed to a certain ion mass (see Supplementary Figure 1). We then multiply the raw data with the masking filter, which yields the isolated mass peak, over which we integrate to obtain the peak signal. The advantage of this slightly complicated procedure lies in the accurate description of bands that may deviate from ideal Gaussians due to ringing effects and other distortions (see Supplementary Figure 1). We perform this procedure on multiple TOF spectra to obtain the average peak signal and its corresponding error.

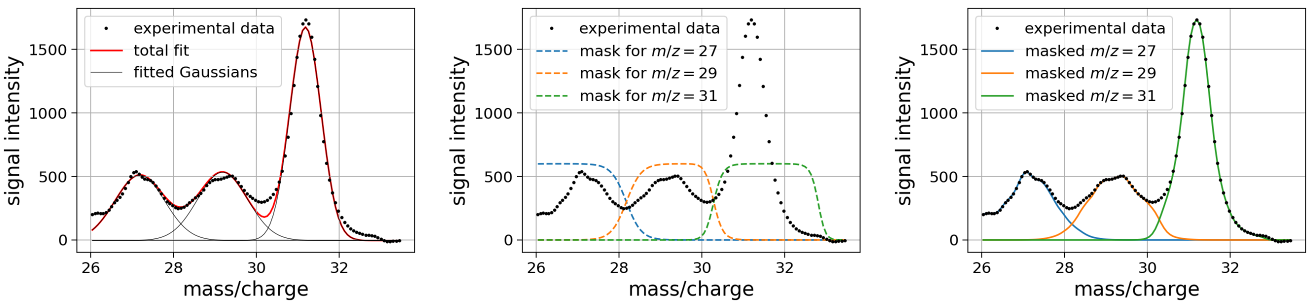

(a) **(b)** **(c)**

Supplementary Figure 1. Example of separating mass peaks $m/z = 27, 29, 31$ through masking. (a) Simultaneous fit of three Gaussians on the TOF spectrum (b) Individual masks for each mass peak (c) Resulting masked peaks.

3 XUV intensity dependence

Data normalization The peak intensities from the XUV pulse are calculated assuming a Gaussian beam with a measured spot size FWHM of 52 μm $\times$ 61 μm, an XUV-UV temporal cross-correlation measurement of 119 fs, and an assumed temporal FWHM of the UV pulse of 100 fs, which yields a temporal FWHM of the XUV of 54 fs.

The raw data for the Ne^+ photoion intensity-dependence measurements show deviations from ideal linear behaviour, and the other ions show similarly correlated deviations as well (Supplementary Figure 2). These deviations do not seem to originate from the detected ion signals, but rather from the intensity monitor which measures the pulse energy of the FEL radiation. To correct for this, we normalize the intensity-dependent ion data by assuming perfectly linear behaviour for the Ne^+ with respect to the XUV pulse energy. We use this to correct the XUV pulse energies of the other ions.

Deviations from expected behaviour The XUV pulse energy dependence of various ion yields are shown in Supplementary Figure 3. $H^+_{1,2,3}$ are presumably purely dicationic products, and are expected to show a quadratic behaviour with respect to the XUV pulse energy, assuming two-photon double ionization is the dominating process. We notice that, at low XUV pulse energy, the parent cation exhibits non-linear behaviour (Supplementary Figure 3d), whereas it is expected to be linear as a one-photon ionization process. We attribute this to the minor presence of ethanol clusters in this intensity-dependence measurement. At higher XUV pulse energies, we see that the parent cation yield saturates, while the $H^+_{1,2,3}$ ion signals show a significant increase. This strong increase is attributed to the non-linearity of the two-photon double-ionization process of ethanol. We note that in the main text, the datasets presented do not have any contribution from clusters.

4 Fitting of pump-probe delay-dependent ion yields

The fit model employed for the pump-probe delay-dependent (t_P) yield of H_3^+ or D_3^+ ions consists of three contributions, as discussed in the main text. The positive enhancement around zero delay was fitted as a Gaussian cross-correlation feature

$$y_C(t_P;\sigma) := a_C e^{\frac{1}{2}\left(\frac{t_P - t_0}{\sigma_C}\right)^2}, \tag{1}$$

where $\sigma_C := \sqrt{\sigma_X^2 + \sigma_U^2} =$119 fs FWHM corresponds to the experimentally measured cross-correlation, t_0 is the zero-delay position between the two pulses, and a_C is a fitted amplitude.

The additional step function, which is used to account for signal differences at long negative and long positive delays ($a_{\pm\infty}$), has the form,

$$y_O(t_P) := \frac{a_\infty - a_{-\infty}}{2} \operatorname{erf}\left\{\frac{t_P}{\sigma_C\sqrt{2}}\right\} - a_{-\infty}. \tag{2}$$

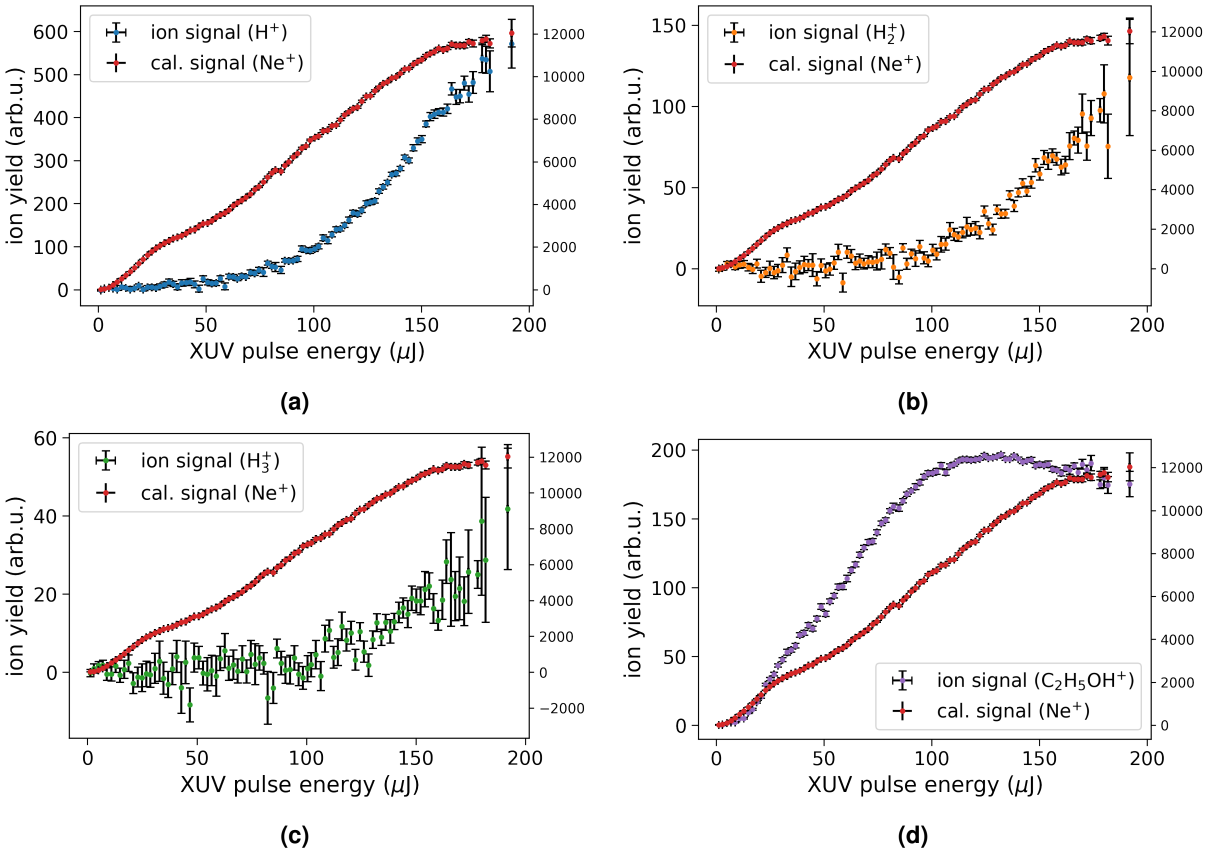

Supplementary Figure 2. Raw intensity dependence of the (a) H^+ (b) H_2^+ (c) H_3^+ and (d) parent $C_2H_5OH^+$ ion yields on the XUV (31.7 eV) intensity.

The exponentially decaying depletion feature y_D is a convolution between a Gaussian and a step function with an exponential decay. For example, the depletion of the H_3^+ ion, originating from double photoionization is found as

$$y_D(t_P;\sigma) \approx s(t;\sigma) * \theta(t) e^{-Rt} \tag{3}$$

where $\theta(t)$ is a step function, and "$*$" denotes convolution, $s(t;\sigma_D)$ is a Gaussian resulting from convoluting three other Gaussians (two from XUV corresponding to two-photon double ionization, and one from UV), with a corresponding width fixed as $\sigma_D = \sqrt{2\sigma_X^2 + \sigma_U^2}$ =135 fs FWHM. The convolution evaluates as

$$y_D(t_P;\sigma) \approx 1 + \frac{1}{2}(C-1)e^{\frac{1}{2}(R\sigma)^2} e^{-Rt_P} \operatorname{erfc}\left\{\frac{R\sigma^2 - t_P}{\sqrt{2}\sigma}\right\}, \tag{4}$$

where C is interpreted as the depletion strength (e.g. $C = 1$ is no depletion, $C = 0$ is total depletion, $C = 2$ is a two-fold enhancement), and erfc is the complementary error function. This "simple" form holds as long as the depletion is small and probe pulses are short. In the case of strong depletion by long probe pulses, saturation of the probe effects may lead to failure of this approximation. Nevertheless, only the apparent time-zero t_0 and convolution width σ would be affected; the rate constant R remains unchanged.

The overall fit for H_3^+ is then

$$y(t_P) = y_O(t_P;\sigma_C) + y_C(t_P;\sigma_C) + y_D(t_P;\sigma_D). \tag{5}$$

Mass fragments other than H_3^+ were fitted with a similar form, but using the cross-correlation of XUV+UV for all contributions, rather than the XUV+XUV+UV cross-correlation of H_3^+,

$$y(t_P) = y_O(t_P;\sigma_C) + y_C(t_P;\sigma_C) + y_D(t_P;\sigma_C). \tag{6}$$

For all fragments other than H_3^+ and D_3^+, which presumably originate dominantly from single photoionization, we let the width σ_D fit freely.

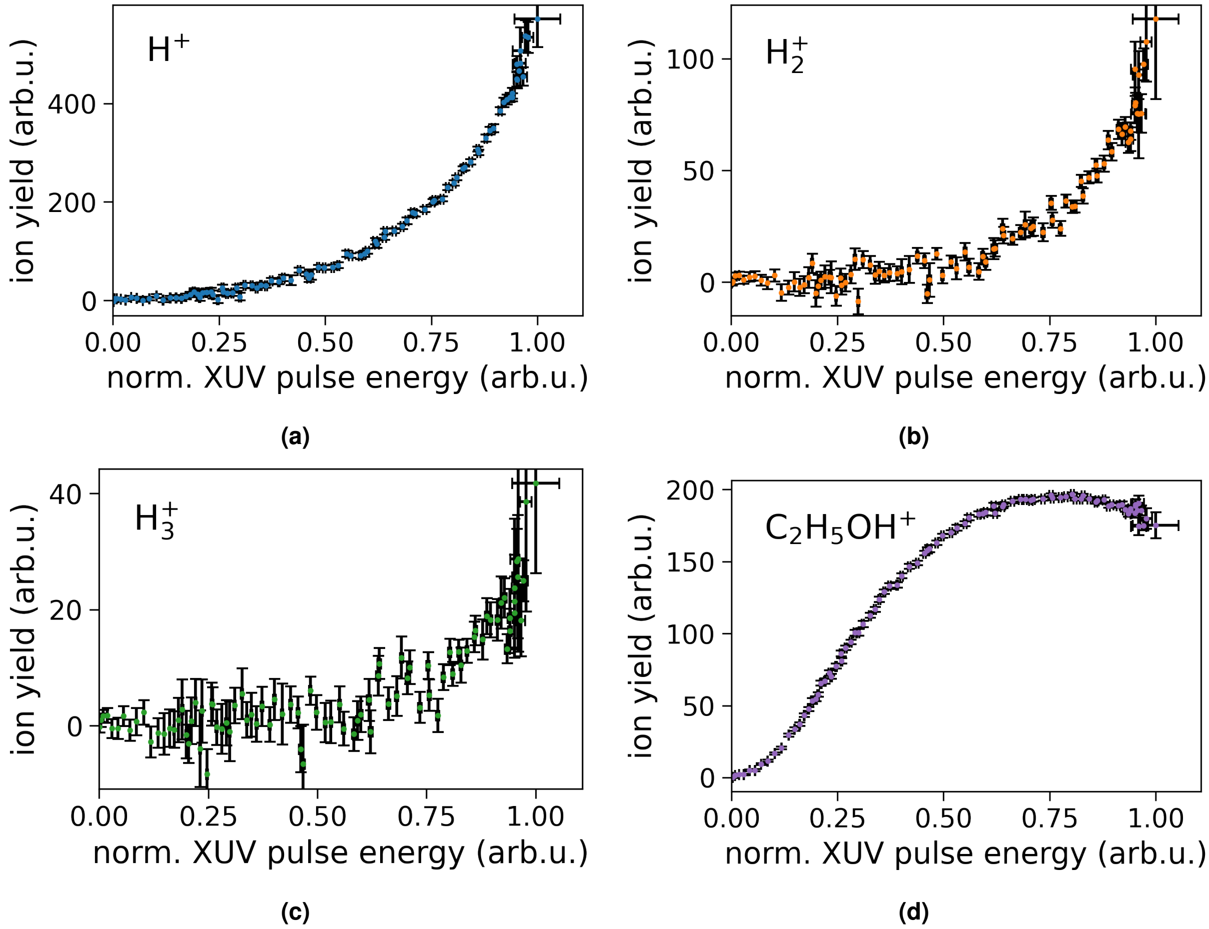

Supplementary Figure 3. Intensity dependence of the (a) H^+ (b) H_2^+ (c) H_3^+ and (d) parent $C_2H_5OH^+$ ion yields on the XUV (31.7 eV) intensity. XUV pulse energy normalized to the Ne^+ signal level, assuming linear dependence of Ne^+ photoionization at 31.7 eV up to $3 \cdot 10^{13}$ W/cm^2. Global error of $\sim$10% for the XUV pulse energy.

Rate equation In the case where the approximation fails, and precise values of t_0 and σ are required, one must consider the system of rate equations whose approximation leads to Eq. (4). We will introduce this here. The disruptive pathway is determined by the cross-section for the XUV-induced double-ionization S, the relaxation time-constant R from the dication state to the roaming product, and the disruption strength D of the UV probe on the roaming process. To describe it in detail, we give the form of our XUV pump $x(t)$ and UV probe $u(t)$ pulses with a pump-probe delay t_P between them,

$$\begin{aligned} x(t) &= \frac{P_x}{\sigma_X\sqrt{2\pi}} e^{-\frac{1}{2}\left(\frac{t}{\sigma_X}\right)^2} \\ u(t;t_P) &= \frac{P_u}{\sigma_U\sqrt{2\pi}} e^{-\frac{1}{2}\left(\frac{t-t_P}{\sigma_U}\right)^2}, \end{aligned} \tag{7}$$

and now form a system of rate equations to describe the time-evolution as:

$$\begin{aligned} y_0'(t) &= -S_1 x(t) y_0, \\ y_1'(t) &= S_1 x(t) y_0(t) - S_2 x(t) y_1(t), \\ y_2'(t) &= S_2 x(t) y_1(t) - D u(t;t_P) y_2(t) - R y_2(t), \\ y_D'(t) &= R y_2(t), \end{aligned} \tag{8}$$

where we have the populations of: the ground state y_0, the cationic state y_1, the undissociated dicationic state y_2, and the H_3^+ product y_D; and the couplings: ionization rate S_1 of y_0 by $x(t)$, ionization rate S_2 of y_1 by $x(t)$, depletion rate D of the y_2 by

Supplementary Table 2. Relative ion yields for 2-aminoethanol (C_2H_7NO) upon XUV ionization.

m/z from C_2H_7NO	possible identity	relative yield at $h\nu = 21.1$ eV	relative yield at $h\nu = 31.7$ eV
1	H^+	55.7(4)	1550(10)
2	H_2^+	22.0(3)	164(4)
3	H_3^+	0.0(4)	13(2)
13	CH^+	2.9(2)	154(2)
14	CH_2^+/N^+	6.7(1)	279(3)
15	CH_3^+/NH^+	265.8(8)	831(6)
17	OH^+/NH_3^+	85.1(5)	373(4)
18	H_2O^+/NH_4^+	272(3)	664(5)
19	H_3O^+	58.9(3)	1073(8)
28	NH_2C^+	372(2)	595(4)
30	CH_4N^+	3173(8)	3150(20)
42	$C_2H_4N^+$	226.2(7)	278(2)
45	$CH_3CH_2O^+$	28.3(3)	43.1(7)
61	$C_2H_7NO^+$	100	100

$u(t;t_P)$, and decay rate R of y_2. The "depletion" contribution is given as $y_D := \lim_{t\to\infty} y_D(t)$,

$$y_D = R\int_{-\infty}^{\infty} dt \left\{ e^{-Du(t;t_P)-Rt} \int_{-\infty}^{t} dt' \left\{ e^{Du(t';t_P)+Rt'} Bx(t') \left(\frac{1}{B-A} e^{-Ax(t')} - e^{-Bx(t')} \right) \right\} \right\}. \quad (9)$$

A closed form for Eq. (9) does not exist, so it must either be numerically evaluated, or approximated (e.g. Eq. (4)).

5 Ion yield data for 2-aminoethanol

Analogous to ethanol in the main text, we provide relative ion yields from photoionization of 2-aminoethanol in Supplementary Table 2.

6 Depletion data for ethanol and 2-aminoethanol

For non-deuterated ethanol, we show a variety of mass fragments at XUV photon energies of 31.7 eV (Supplementary Figure 4) and 24.7 eV (Supplementary Figure 5). For fully-deuterated ethanol, mass fragments obtained at an XUV photon energy of 31.7 eV are shown in Supplementary Figure 6. For 2-aminoethanol, we have obtained fragment mass at XUV photon energies of 31.7 eV (Supplementary Figure 7) and 21.1 eV (Supplementary Figure 8).

7 Depletion data for Ne^+ (m/z=20, 22) and Ne^{2+} (m/z=10)

For the measurements performed on ethanol, we additionally include data for the Neon ion yields $^{20}Ne^+$, $^{22}Ne^+$, and $^{20}Ne^{2+}$, (Supplementary Figures 9, 10 and 11). In a previous study[1] the Ne^{2+} yield has been used to determine the cross correlation between a broad band XUV pump pulse and an NIR probe pulse. Our data show that the variation of the experimental conditions between measurements (see Supplementary Table 1) influence the observed dynamics considerably. For the measurements on H_6-ethanol with 31.7 eV, the Ne^{2+} signal is enhanced by a delayed UV pulse, that induces multi-photon ionization of excited states of Ne^+. The corresponding depletion of the Ne^+ signal is only observed for the ^{22}Ne isotope, since the more abundant ^{20}Ne isotope slightly saturated the detector. In the data set on D_6-ethanol at the same photon energies (Supplementary Figure 11) the lower UV intensity (see Supplementary Table 1) makes these multi-photon ionization processes unfeasible, and thus explains the lack of dynamics in the neon ion yields. For the measurement at 24.7 eV (Supplementary Figure 10) the different XUV photon energy changes the populated excited states of Ne^+ and relative yields of Ne^+ and Ne^{2+}. Here only the Ne^+ signals show dynamics in their yields.

References

1. Livshits, E., Luzon, I., Gope, K., Baer, R. & Strasser, D. Time-resolving the ultrafast H_2 roaming chemistry and H_3^+ formation using extreme-ultraviolet pulses. *Commun. Chem.* **3**, 1–6, DOI: 10.1038/s42004-020-0294-1 (2020).

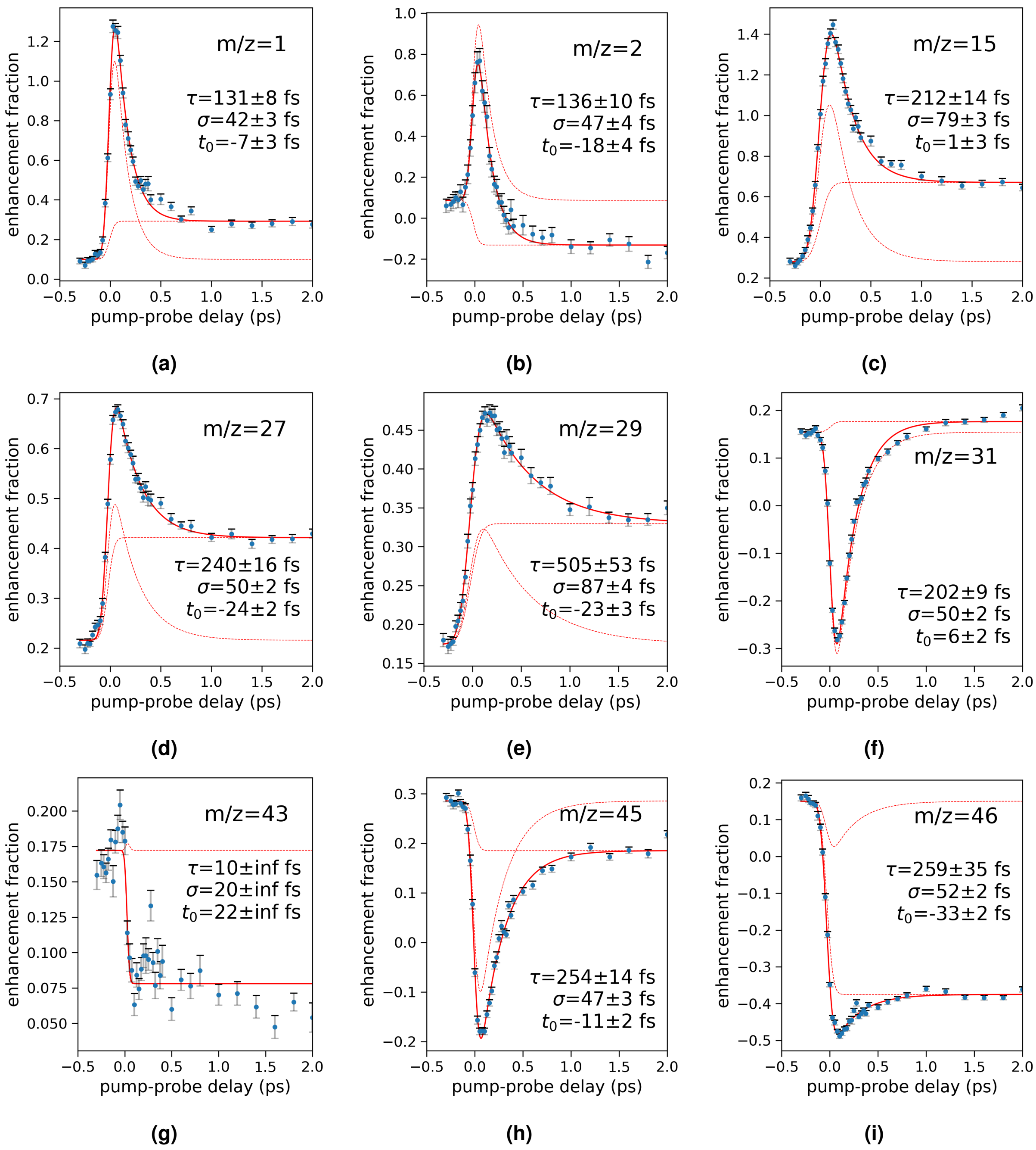

Supplementary Figure 4. Pump-probe-delay-dependent ion yields from the photoionization of non-deuterated ethanol, at an XUV photon energy *hν*=31.7 eV. The data was fit by the model in Eq. (5).

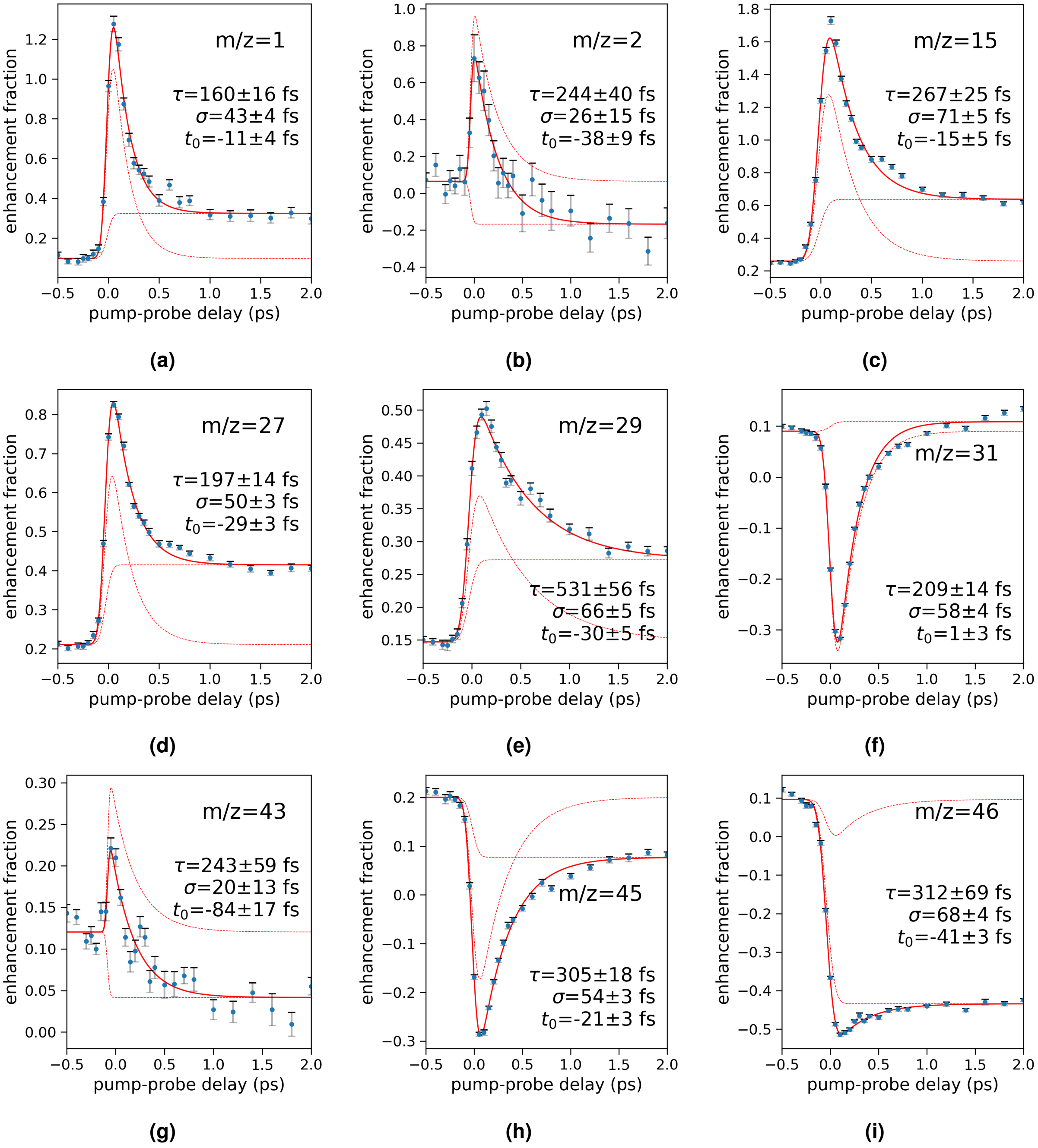

Supplementary Figure 5. Pump-probe delay-dependent ion yields from the photoionization of non-deuterated ethanol, at an XUV photon energy *hv*=24.7 eV. The data was fit by the model in Eq. (5).

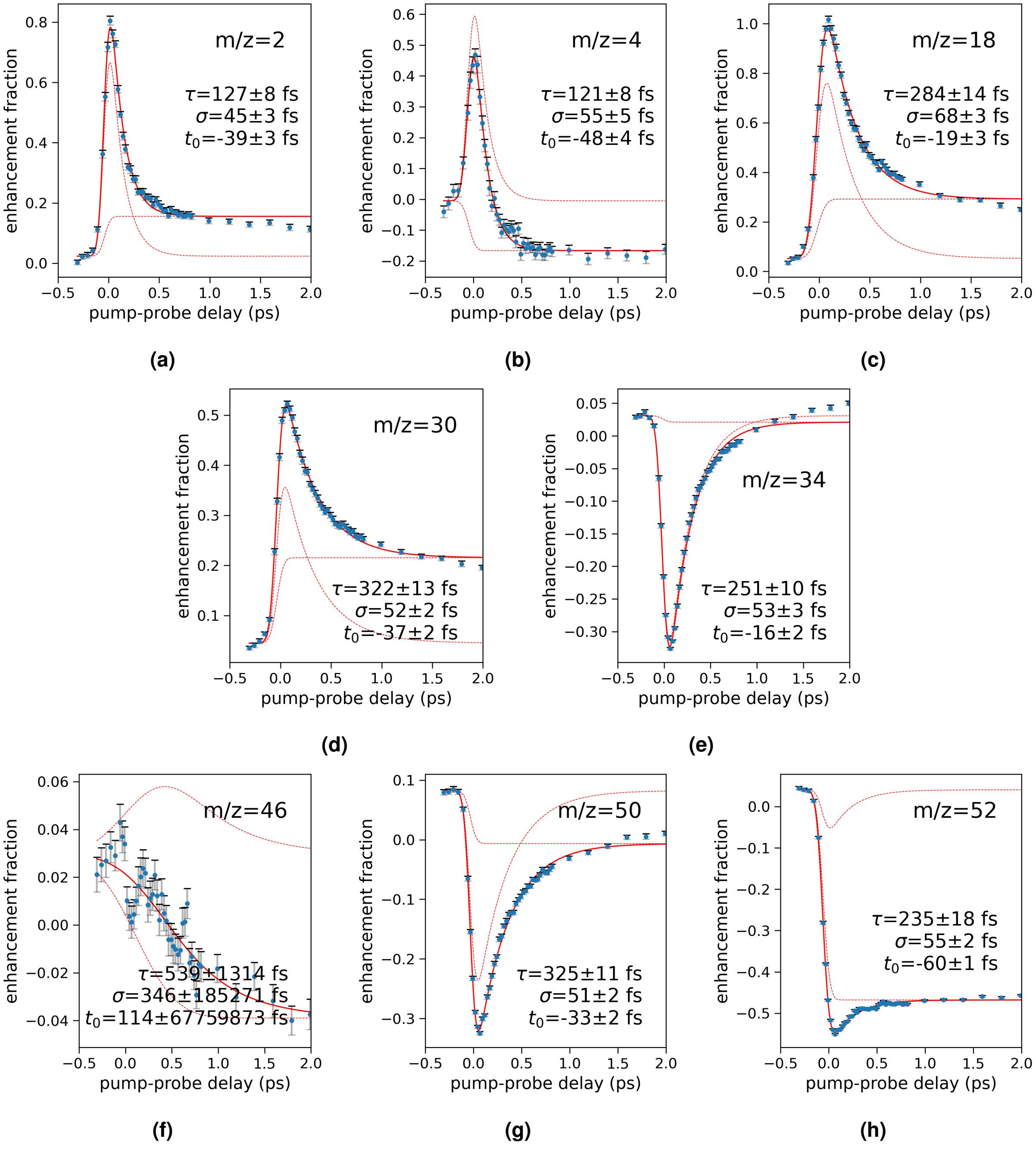

Supplementary Figure 6. Pump-probe delay-dependent ion yields from the photoionization of fully-deuterated ethanol, at an XUV photon energy $h\nu$=31.7 eV. The data was fit by the model in Eq. (5).

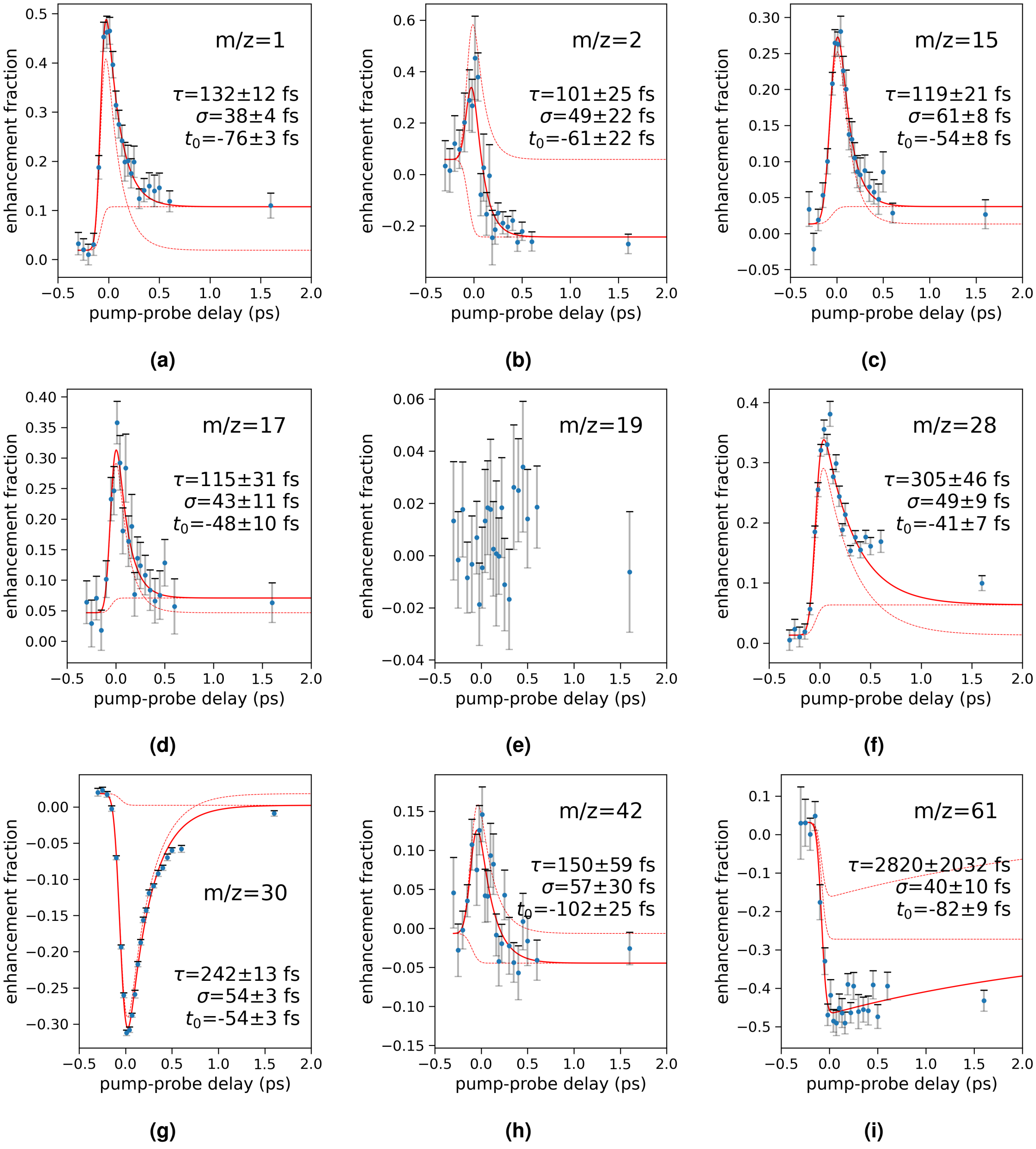

Supplementary Figure 7. Pump-probe delay-dependent ion yields from the photoionization of non-deuterated 2-aminoethanol, at an XUV photon energy $h\nu$=31.7 eV. The data was fit by the model in Eq. (5).

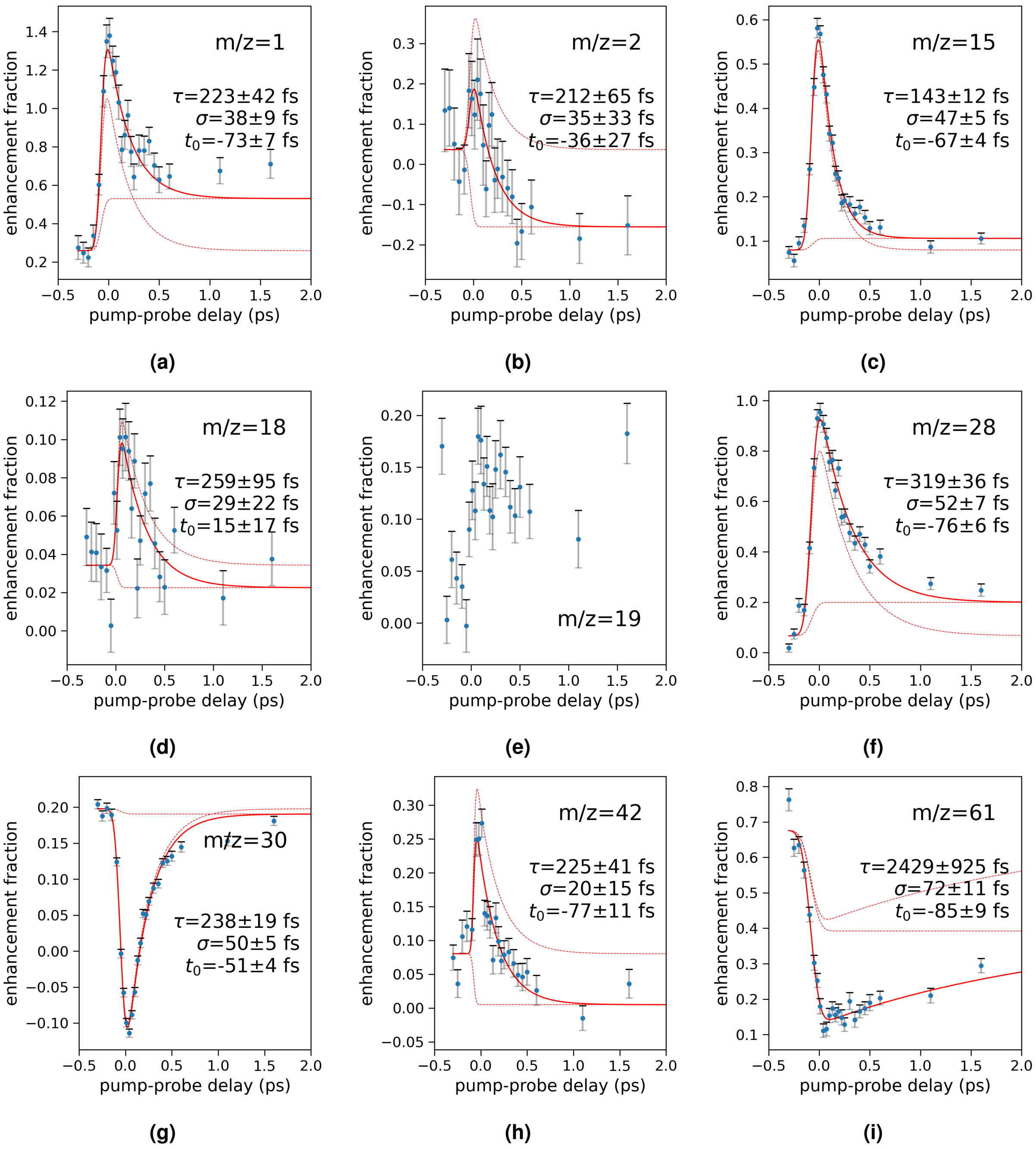

Supplementary Figure 8. Pump-probe delay-dependent ion yields from the photoionization of non-deuterated 2-aminoethanol, at an XUV photon energy $h\nu$=21.1 eV. The data was fit by the model in Eq. (5).

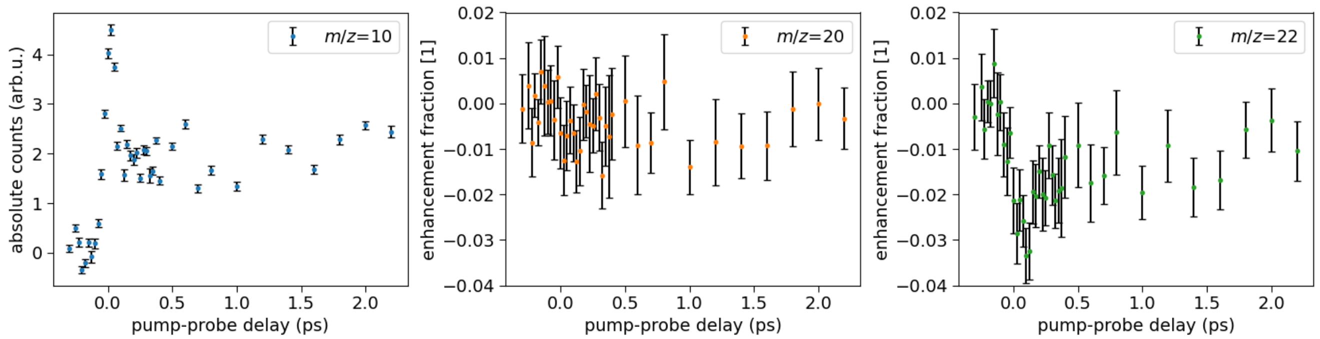

Supplementary Figure 9. Pump-probe delay-dependent ion yields of Neon obtained concurrently with non-deuterated ethanol (c.f. Supplementary Figure. 4, at an XUV photon energy $h\nu$=31.7 eV. Absolute ion-yield shown for m/z=10, while the enhancement ratio for m/z=20, 22 are given.

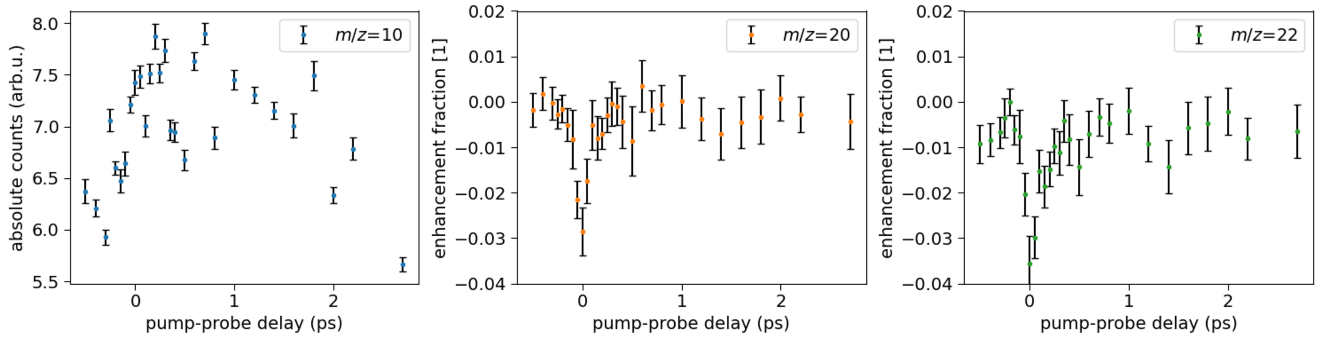

Supplementary Figure 10. Pump-probe delay-dependent ion yields of Neon obtained concurrently with non-deuterated ethanol (c.f. Supplementary Figure. 5, at an XUV photon energy $h\nu$=24.7 eV. Absolute ion-yield shown for m/z=10, while the enhancement ratio for m/z=20, 22 are given.

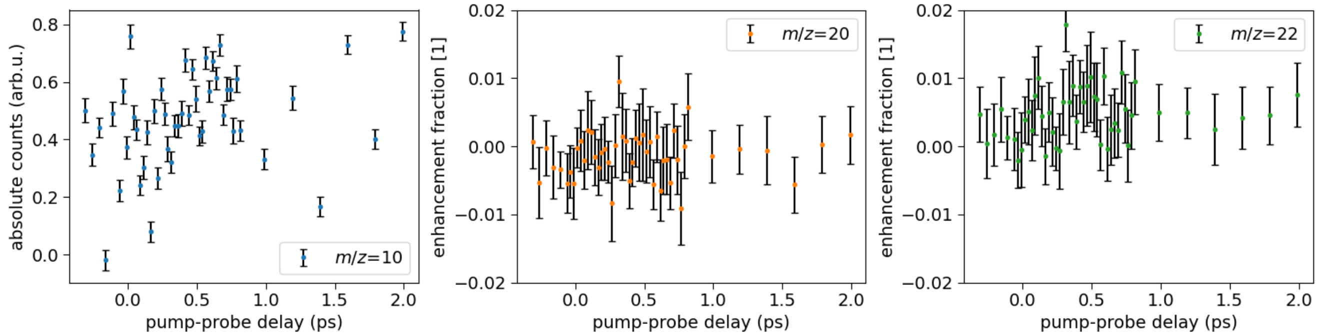

Supplementary Figure 11. Pump-probe delay-dependent ion yields of Neon obtained concurrently with fully-deuterated ethanol (c.f. Supplementary Figure. 4, at an XUV photon energy $h\nu$=31.7 eV. Absolute ion-yield shown for m/z=10, while the enhancement ratio for m/z=20, 22 are given.
